# An overview of existing modeling tools making use of model checking in the analysis of biochemical networks

**DOI:** 10.3389/fpls.2012.00155

**Published:** 2012-07-20

**Authors:** Miguel Carrillo, Pedro A. Góngora, David A. Rosenblueth

**Affiliations:** Departamento de Ciencias de la Computación, Instituto de Investigaciones en Matemáticas Aplicadas y en Sistemas, Universidad Nacional Autónoma de MéxicoMéxico D.F., México

**Keywords:** model checking, gene regulatory networks, biochemical networks, model analysis, complex systems

## Abstract

Model checking is a well-established technique for automatically verifying complex systems. Recently, model checkers have appeared in computer tools for the analysis of biochemical (and gene regulatory) networks. We survey several such tools to assess the potential of model checking in computational biology. Next, our overview focuses on direct applications of existing model checkers, as well as on algorithms for biochemical network analysis influenced by model checking, such as those using binary decision diagrams (BDDs) or Boolean-satisfiability solvers. We conclude with advantages and drawbacks of model checking for the analysis of biochemical networks.

## 1. Introduction

A basic conviction in computational biology is that it should be possible to create computational tools allowing us to considerably increase our understanding of the functional properties of living organisms. *Model checkers* are mainly used in the design of digital circuits and stand out as computational tools especially successful in the analysis of complex systems. Hence, it is unavoidable to consider the applicability of model checking to computational biology (Fisher and Henzinger, 2007). In fact, model checking has already been incorporated into a number of computer systems for the analysis of biochemical networks. Our purpose is to review several tools that use model checking in the analysis of biochemical networks, so as to assess the potential of model checking in computational biology.

### 1.1. Model checking

Model checking is a verification technique allowing us to determine whether or not a system model meets a specification. As compared with other verification techniques, model checking has a number of features making it an industrial-strength methodology. Model checking is, for example, routinely used in the design of integrated circuits. Model checking inventors, moreover, were distinguished with the A. M. Turing award in 2007.

### 1.2. Graph search and branching time

The verification process in a model checker often uses a graph-search algorithm accumulating, in a set, system states having a desired property. Model checkers typically do not represent the elements of such sets explicitly, but implicitly, with techniques named *symbolic*. A symbolic model checker can often represent sets with a vast number of states.

Graph-traversal in model checkers should be contrasted with that of simulators. A simulator visits system states in the *same* order as states occur in the simulated system. Model checkers, by comparison, normally traverse the state graph in *reverse*, starting from states which trivially have a property of interest and proceeding backwards toward the rest of the states. This difference has advantages when the model of the system has states with *more than one successor*. The reason is that these models may have infinitely many trajectories, so that traversing all such trajectories forward, as a simulator does, would be infeasible.

The possibility of having more than one successor for a state translates to *branches* in time, a feature often appearing in models analyzed by model checkers. Branching time can model a variety of important phenomena, such as the interaction with the environment. A reason is that because the behavior of the environment is not determined, and the next state of the system being analyzed partially depends on the environment, the next state of such a system is not completely determined either. Other phenomena, such as asynchrony and incomplete knowledge of a network can be modeled with branching time as well (Thomas and D'Ari, 1990).

It might seem at first sight that model checking is an ordinary method for exhaustive graph-traversal. Such methods are often shunned as they are subject to the “state-explosion problem” and can readily become intolerably inefficient as the size of a network increases. Although model checkers do perform exhaustive search (Emerson, 2008), symbolic methods often override the state-explosion problem: symbolic representations are in many cases surprisingly concise, yielding efficient traversals. Moreover, the verification of some systems can *only* be tackled symbolically (Bloem et al., 2000).

### 1.3. Beyond the original model checking

Model checking is most often employed in the analysis of state-transition systems. The reason is that initially model checking was applied to “Kripke structures,” which can be regarded as such systems. Variables in such structures are Boolean, and time is discrete. Model checking, nonetheless, has since been extended to numerous other kinds of models. For example, by adding probabilities, a Kripke structure can be regarded as either a discrete- or continuous-time Markov chain or even a Markov decision process. Logics and algorithms have been developed for model-checking such processes (Hansson and Jonsson, 1994; Bianco and de Alfaro, 1995; Baier et al., 2003; Kwiatkowska et al., 2005). By adding continuous variables, a Kripke structure can be viewed as a hybrid automaton (Alur et al., 1993; Henzinger et al., 1997). Continuous time for automata can also be modeled, with timed automata (Alur and Dill, 1994).

Therefore, although the most direct use of model checking in biochemical networks would be in discrete models, other kinds of model are also potentially amenable to be model-checked.

### 1.4. Model checking in biochemical networks

In the sequel, we will encounter model checking employed in various ways for the analysis of biochemical networks. Perhaps the kind of model that is most often used for such networks is a set of differential equations. If ordinary model checking is chosen, however, the gap between such continuous models and discrete state-transition systems must be bridged.

On a different dimension, we will see that many computer tools for biochemical network analysis employ model checking for verifying that a model has a desired property (as is usually done in other domains, like digital circuits). Other systems, nevertheless, are able to extract more information from a model checker. For instance, by forcing a model checker to compute a counterexample, it is possible to obtain a path with a certain property. Another way is to have the model checker report all the states having a specified property.

Finally, we will see examples of less usual kinds of model checking, like probabilistic and hybrid model checking.

### 1.5. Structure of the paper

After reviewing models of biochemical networks in section 2, we turn our attention to model checking in section 3. Section 4 summarizes the use of model checking in tools for biochemical network analysis, while section 5 is devoted to works reporting direct applications of model checkers. Section 6, by contrast, describes computer tools or isolated algorithms that do not necessarily employ full-fledged model checking, but do use a symbolic technique. Section 7 draws some conclusions.

## 2. Models of biochemical networks

From the point of view of model checking, there is no essential difference, in terms of analysis with this technique, between various kinds of either biochemical networks or GRNs. We, therefore, use “biochemical network” to refer to several families of networks, such as gene, metabolic, signal-transduction, and cell-cycle networks (Deville et al., 2003).

### 2.1. Gene regulatory networks

A GRN is a collection of DNA fragments indirectly interacting with each other and controlling the transcription of genes into mRNA. In the study of GRNs, analytical approaches represent the more realistic end of the model spectrum. Such models consist of nonlinear systems of ordinary differential equations (ODEs), where each variable denotes the concentration of a different gene product. Non-linearities, often modeled with sigmoids, appear from the fact that often the concentration of a product changes non-linearly with respect to another one. These non-linearities create mathematical difficulties, even for finding the set of attractors. A simplification of such models approximates sigmoids by step functions, giving rise to stepwise-linear equations (Gouzé and Sari, 2002). Such treatments have the advantage of being amenable to qualitative analysis of steady-state and transient behavior of regulatory systems (de Jong et al., 2004). Next in the spectrum would be models of Thomas' formalism (Thomas and D'Ari, 1990). Such models are multi-valued state-transition systems. Activation levels of genes are represented with discrete variables and time is viewed as proceeding in discrete steps. The value of every gene *x* at time *t* + 1 is specified by a function of the values of its regulators *y*_1_, *y*_2_, … , *y*_*n*_*x*__ at time *t*. Boolean GRNs (Kauffman, 1969) are Boolean state-transition systems, where each gene has only two possible activation values: active (1) or inactive (0); intermediate expression levels are neglected. A *network state* at time *t* is a vector containing the activation values of all the genes in the GRN at time *t*. Time is also discrete. The value of every gene *x* at time *t* + 1 is specified by a function of the values of its regulators *y*_1_, *y*_2_, … , *y*_*n*_*x*__ at time *t*. Finally, an interaction graph can be considered an even more abstract model of a biochemical network (Fages and Soliman, 2008b). We refer the reader to the review (de Jong, 2002).

### 2.2. Metabolic pathways

Metabolic pathways are series of chemical reactions catalized by enzymes, often employing vitamins and other dietary substances, termed metabolites. Metabolites are modified through formation and dissolution of chemical bonds. Non-probabilistic models can be adequate because unstable equilibria are rare and large numbers of molecules are present (Bower and Bolouri, 2001).

### 2.3. Signal-transduction pathways

Signal transduction refers to the transfer of information (called signals) from the extracellular medium, first to the cell membrane, and then to the intracellular medium, causing a response. By comparison with metabilic pathways, signal transduction pathways present a more complex dynamics with small numbers of relevant molecules. This makes probabilistic models more appropriate for such networks (Bower and Bolouri, 2001).

### 2.4. Cell-cycle networks

The cell cycle is the series of phenomena happening when a cell grows, divides, and duplicates. Models of cell-cycle networks also range from differential equations (Chen et al., 2004) to Boolean networks (Davidich and Bornholdt, 2008).

## 3. Model checking

Model checking (Clarke and Emerson, 1981; Quielle and Sifakis, 1981) can be regarded as an instance of the *verification problem*: determining whether or not a given computer program meets a given specification. Initial research on verification (Floyd, 1967) concentrated on finite computations of sequential programs. The desire, a decade later, for dealing with infinite computations and with concurrent programs motivated the development of new techniques, based on *temporal logic*, where the truth of statements can vary in time (Pnueli, 1977). Temporal logics also allowed reasoning about “reactive” systems, having an ongoing interaction with the environment. The unpredictability of the environment appears as *branching time*, where a state can have more than one possible future. Non-terminating computations are reflected as infinite sequences of states. Hence, the semantics of a reactive system can be given as an infinite tree of states. In spite of such trees being infinite, the number of states may be finite, allowing the application of efficient graph-traversal methods.

Unlike other verification methods, model checking is totally automatic, and the specification is formulated in mathematical (temporal) logic. In addition, model checking not only deals with correctness but also with incorrectness, often providing a *counterexample* in case the program does not meet the specification.

Expressiveness of logics used in model checking, however, should be limited to achieve good performance. Hence, (temporal) logic is sometimes restricted so that only a partial behavior of the system may be specified. In this sense, model checking is a weak version of the verification problem. In spite of this, restricted temporal logics can express, among others, liveness properties, e.g., “every request will eventually be granted,” and safety properties, e.g., “certain state will never be reached” (Emerson, 2008).

An important breakthrough in model checking was the development of “symbolic” techniques, where states are represented implicitly. The first of these techniques was the introduction (Burch et al., 1992) of reduced, ordered binary decision diagrams (BDDs). A BDD is a representation of a Boolean function, where all redundancy has been removed. Such a representation can be seen as a graph in which a polynomial number of nodes may have an exponential number of paths. Since each path corresponds to a state, such a graph can represent a large number of states. Another symbolic technique is the codification of a model-checking problem as the satisfiability of a Boolean expression (SAT). This method employs algorithms for solving the SAT problem resulting from the phenomenal recent progress in the development of SAT solvers.

The main problem model checking faces is that of “state explosion,” as the size of the model increases *exponentially* in the number of parameters of the model. A notable achievement is precisely that “Despite being hampered by state explosion, […] model checking has had a substantive impact on program verification efforts” (Emerson, 2008). “Although the worst-case time complexity of symbolic algorithms is typically worse than that of corresponding explicit algorithms, they perform well as heuristics, so that many large problems can only be tackled symbolically” (Bloem et al., 2000).

It must be emphasized that not all systems with, say 10^90^, states can be handled (Emerson, 2008). Nonetheless, symbolic methods often work on the large systems encountered in practice.

### 3.1. Computation-tree logic

We illustrate model checking for a logic ℒ, by using the case of ℒ = CTL. More thorough treatments are in: (Clarke et al., 1999; Bérard et al., 2001; Huth and Ryan, 2004; Baier and Katoen, 2008).

#### 3.1.1. Kripke structures

**Figure 1:**
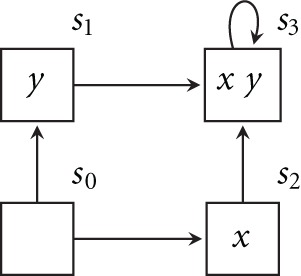
**A Kripke structure**.

Truth of CTL formulas is defined in Kripke structures (also called Kripke models). Figure 1 illustrates one such structure, consisting of: (1) a set *AP* of Boolean variables, i.e., atomic propositions ({*x*, *y*}), (2) a set *S* of states ({*s*_0_, … , *s*_3_}), each labeled with a subset of *AP*, and (3) an *accessibility relation* over *S* which is *serial*, i.e., every state has (at least) an outgoing transition. A *path* starting at a state *s*_0_ is an infinite sequence *s*_0_ *s*_1_ … of states such that *s*_*i*_ and *s*_*i*+1_ are related by the accessibility relation.

#### 3.1.2. Formulas

CTL formulas can have Boolean operators, such as **not** (¬), **or** (∨), **and** (∧), implication (→), and equivalence (↔). In addition, such formulas can have temporal operators, allowing us to refer to formulas holding in the future of a particular state (when interpreting the accessibility relation as time). In this case, we must indicate whether we mean some future or all futures. Hence, we must refer either (1) to *some* path starting in the current state with the existential “modality” **E**, or (2) to *all* paths starting in the current state with the universal modality **A**. Similarly, we can refer (a) to the immediate future with the modality **X**, (b) to some state in the future (including the present) with the modality **F**, or (c) to all states in the future (including the present) with the modality **G**. The following table summarizes these modalities.

**Table:** 

**Modality**	**Meaning**
**E**	Some path (i.e., there Exists a path)
**A**	All paths
**X**	NeXt state (i.e., immediate future)
**F**	Some state either in the present or in the Future
**G**	All states in the present and in the future (Global)

A CTL *temporal operator* is composed of a modality in the upper part together with a modality in the lower part of this table, resulting in six temporal operators. For example, a formula asserting that there exists a path such that in the present or in the future *x* does not hold and *y* does hold would be: “**EF** ((**not** *x*) **and** *y*).”

Often more temporal operators are included in CTL. For example, a generalization of “**EF** β,” written “**E**[α **U** β]” (for “Exists Until”), holds when β holds in the present or in the future, traversing only states in which α holds. Hence, if a formula “σ_*i*_” holds only at a state *s*_*i*_, then “**not E**[(**not** σ_1_) **U** σ_2_]” expresses that it is necessary to go through a state *s*_1_ to reach a state *s*_2_. Such a formula asserts (equivalently) that there does not exist a path that can reach *s*_2_ without reaching *s*_1_.

#### 3.1.3. Model checking algorithm

Normally, an ordinary CTL model checker follows the “state-labeling” algorithm (Clarke et al., 1986). This algorithm traverses a state-transition graph backwards, gradually accumulating, in a set, the states satisfying the desired property. Consider, for example, a liveness property of an elevator model: “every request is eventually granted,” i.e., “every time a button is pressed on a floor will cause the elevator to eventually arrive at such a floor.” Following the state-labeling algorithm, a CTL model checker first computes all the states in which a request has already been granted (i.e., where the elevator has already arrived). This set of states can be trivially computed, as is part of the representation of the system. Then the model checker traverses the state-transition graph backwards, adding states capable of reaching any state in the current set in one time step. The model checker repeats this process until the set stops growing. Such a set will have exactly all the states from which every request is eventually granted.

### 3.2. Other logics

In addition to CTL, linear-time temporal logic (LTL) is often employed in practice. LTL also uses Kripke structures, but the temporal operators of this logic lack the **E** and **A** modalities. Hence, truth of an LTL formula is defined with respect to a single path. A formula holds at a state of a Kripke structure if such a formula holds with respect to every path starting at that state.

When model checking is applied to biochemical network analysis, there are often interesting properties that cannot be expressed in CTL or LTL. For example, neither of these logics can specify the states from which it is possible for a Boolean variable to oscillate (i.e., to switch infinitely many times back and forth between 0 and 1). To be sure, there exists a CTL formula, namely “**EG** ((*x* → **EF not** *x*) **and** (**not** *x* → **EF** *x*))”, approximating oscillations. This formula is necessary but not sufficient for oscillations (which may or may not be adequate for a particular kind of analysis). Sometimes, therefore, more expressive logics must be considered.

It is also possible to apply model checking to other kinds of models. A probabilistic version of CTL (PCTL) has been developed (Hansson and Jonsson, 1994; Bianco and de Alfaro, 1995), which employs discrete-time Markov chains or Markov decision processes instead of Kripke structures. Continuous-time Markov chains (CTMC) can be model-checked with continuous stochastic logic (CSL) (Baier et al., 2003). Furthermore, hybrid models (with continuous variables) (Alur et al., 1993) and timed-automata (with continuous time) (Alur and Dill, 1994) can be model-checked with appropriate logics.

## 4. Specialized tools

Biocham and Genetic Network Analyzer (GNA) are perhaps the computer tools for biochemical network analysis most extensively using model checking. We thus start with these two systems, and proceed with SMBioNet, Pathway Logic, Antelope, and XSSYS.

### 4.1. Biocham

#### 4.1.1. Overview

Biocham (BIOCHemical Abstract Machine) (Fages and Soliman, 2008b) can analyze and simulate biochemical networks using differential, stochastic, discrete, and Boolean models. In addition, properties can be formalized in temporal logic (LTL with numerical constraints, probabilistic LTL, and CTL), so that a model checker can be used to validate such properties. Moreover, Biocham can compute the violation degree of LTL formulas. Finally, Biocham has a model-update module, repairing models that do not satisfy CTL properties.

Biocham's models are specified with a set of reaction rules of the form “*e*_*i*_ for *S*_*i*_ => *S*′_*i*_,” *i* = 1, … , *n*, over molecular concentration variables *x*_1_, … , *x*_*m*_, where *e*_*i*_ is a kinetic expression involving the concentration of molecules, *S*_*i*_ is a set of molecules with their stoichiometric coefficient, and *S*′_*i*_ is the transformed set of molecules. Examples of kinetic expressions are: (1) the mass action law kinetics, (2) the Michaelis–Menten kinetics, and (3) the Hill kinetics. A set of such rules defines a (hyper) graph which can be interpreted by Biocham under different semantics.

In the case of a system ODEs semantics, Biocham can simulate, using the Runge–Kutta method or Rosenbrock's method, such systems of equations. In addition, Biocham can interpret rules with a stochastic semantics as a continuous-time Markov chain where the kinetic expressions are transition rates. Simulations in this case can be performed with Gillespie's algorithm (Gillespie, 1976), for example. Next in the abstraction-level progression, a set of rules can be interpreted as an asynchronous discrete model. Biocham obtains such a model from the stochastic semantics by simply disposing of the transition probabilities. As a result, this model employs branching time (but not probabilities). Finally, Biocham can view a set of rules as an asynchronous Boolean network. In this case, Biocham obtains a Boolean network directly from the biochemical reaction rules. Branching time appears because a rule such as A + B => C, for instance, is translated into four transitions going out of the same state, resulting from the four combinations of either reactant A or reactant B being completely or incompletely consumed.

These abstractions, which start from a reaction model and proceed to stochastic, to discrete, to Boolean networks, overapproximate the Boolean semantics obtained from the quantitative semantics. Hence, the non-existence of a behavior in the Boolean semantics implies its non-existence in the quantitative semantics of the rules (Fages and Soliman, 2008b). Fages and Soliman (2008a) use an algebraic theory of abstract interpretation to formalize reaction models and the stochastic, discrete, and Boolean semantics by lattices $D_{ℛ}$, $D_{S}$, $D_{D}$, and $D_{ℬ}$, respectively. Then, the authors prove that such lattices form a hierarchy of abstractions by constructing Galois connections between $D_{ℛ}$ and $D_{S}$, $D_{S}$ and $D_{D}$, and $D_{D}$ and $D_{ℬ}$.

Finally, Fages and Soliman (2008c) have observed that if no molecule is both an activator and an inhibitor of the same target molecule, then the interaction diagram obtained from an ODE model coincides with that obtained from a set of reaction rules. As a result, Biocham can efficiently compute such an interaction diagram simply by a syntactic inspection of the reaction rules.

#### 4.1.2. Model checking in Biocham

Using the NuSMV model checker (Cimatti et al., 2000), Biocham can model-check its Boolean networks with respect to CTL formulas. For example, Biocham abbreviates “**not E**[(**not** *Q*) **U** *P*]” as checkpoint (Q, P). CTL (without “strong fairness”) can also approximate oscillations with necessary, but not sufficient, formulas (seen in subsection 3.2). For many applications such formulas are helpful.

Biocham can use model checking in other forms. First, an extension of ordinary LTL with constraints over reals allows to analyze traces obtained from simulations (Rizk et al., 2011). Essentially, such a logic adds variables to LTL formulas, as in G[A]<x, expressing the constraint that x is always greater than the maximum concentration of A. Currently, Biocham has a model checker for the fragment of such a logic in which linear constraints can appear as atomic propositions. Furthermore, Biocham is able to compute the violation degree of a formula. Intuitively, the violation degree is the distance between a particular behavior of a system, given as a path, and the expected behavior, given as a temporal-logic formula (Rizk et al., 2009). Such a violation measure can be used to estimate a fitness function with evolutionary optimization methods. This is done by finding kinetic parameter values satisfying a set of biological properties formalized in temporal logic. In addition, such a measure can be used to estimate the robustness of a biological model with respect to its temporal specification.

Next, probabilistic model checking is also provided. Biocham estimates the probability of an LTL formula holding by sampling stochastic simulations (Fages and Soliman, 2008b).

Finally, Biocham has an “update” component for automatically modifying a network that does not satisfy a given CTL formula. The algorithm of this component is based on counterexamples computed by NuSMV. Although incomplete (in the sense of sometimes not being able to find the appropriate changes to networks), such a component is useful because of being able to handle large networks (Chabrier-Rivier et al., 2005).

Biocham has been applied (by its developers) to a budding yeast cell cycle model, to the Mitogen-Activated Protein Kinase (MAPK) cascades, and to the mammalian cell-cycle control. This last network involves 732 reactions over 165 proteins and genes, and 532 variables (implying 2^532^ ≃ 10^160^ states) (Chabrier-Rivier et al., 2004, pp. 36, 40). Moreover, Biocham has been used by biologists working independently from Biocham's developers. Bellé et al. (2010), for instance, has used Biocham to model the cap-dependent translation initiation in sea urchin.

### 4.2. GNA

#### 4.2.1. Overview

GNA (de Jong et al., 2004) is based on a piecewise-linear differential-equations approach, allowing qualitative reasoning about GRNs. Qualitative analysis of GRNs is important because the mechanisms governing gene interactions as well as the qualitative information on kinetic parameters and molecular concentrations are often only partially known. The equations employed by GNA were proposed by Mestl et al. (1995), extending work by Glass and Kauffman (1973). The state variables represent concentrations of gene products; the differential equations, in turn, model the regulatory influences. This approach is related to the formalism developed by Thomas and his colleagues (Thomas and D'Ari, 1990): Snoussi (1989) showed that Thomas' formalism (described below) can be regarded as an abstraction of a special case of this model (de Jong et al., 2004).

The equations of this kind of model have the form:

(1) $$\begin{array}{c} {\dot{x}}_{i}=f_{i}(x_{1},\ldots ,x_{n})-g_{i}(x_{1},\ldots ,x_{n})x_{i},\;1\leq i\leq n \end{array}$$

where ${\dot{x}}_{i}$ is the rate of change of protein *x*_*i*_, *f*_*i*_ the rate of synthesis of *x*_*i*_, and *g*_*i*_ the rate of degradation of *x*_*i*_. The rate of synthesis is defined as:

$$f_{i}\left(x_{1},\ldots x_{n}\right)=\sum_{l\in L}{\text{κ}}_{il}b_{il}\left(x_{1},\ldots ,x_{n}\right)$$

where κ_*il*_ is a rate parameter (κ_*il*_ > 0), *b*_*il*_ is a *regulation function* mapping the non-negative reals into {0, 1}, and *L* is a possibly empty set of indices of regulation functions. The rate of degradation is similar:

$$g_{i}\left(x_{1},\ldots x_{n}\right)=\sum_{l\in L}{\text{γ}}_{il}b_{il}\left(x_{1},\ldots ,x_{n}\right)$$

where *g*_*i*_ is strictly positive. Observe that these equations are piecewise-linear.

A regulation function *b*_*il*_ can be defined as an expression of step functions:

$$\begin{array}{cc} s^{+}(x_{j},\theta_{j}) & =\{\begin{array}{cc} 1 & \text{if}\;x_{j}>\;\theta_{j}\\ 0 & \text{if}\;x_{j}<\;\theta_{j} \end{array}\\ s^{–}(x_{j},\theta_{j}) & =1\;–s^{+}(x_{j},\theta_{j}) \end{array}$$

where θ_*j*_ is a threshold. These thresholds divide the *n*-dimensional phase space into *domains* of dimension *n*, separated by the (*n* − 1)-dimensional hyperplanes *x*_*i*_ = θ^*j*^_*i*_ (Gouzé and Sari, 2002). Within each domain, the behavior is linear, whereas on the hyperplanes separating the domains, mathematical complications arise. The reason is that discontinuous right-hand sides may appear in the equations. Gouzé and Sari (2002) applied a technique developed by Filippov (1988), essentially replacing differential equations by differential inclusions.

GNA performs an abstraction of a system of equations of the form (1) by associating such a system with a state-transition graph. In such a graph, each domain of dimension *k* ≤ *n* is identified with a qualitative state. There exists a transition between two qualitative states if some solution trajectories starting in one corresponding domain reach the other corresponding domain, without passing through an intermediate domain (de Jong et al., 2004, p. 314). As a result of replacing equations by inclusions, solutions (in the sense of Filippov) may not be unique (de Jong et al., 2004, pp. 311, 312). Therefore, a state in the state-transition graph may have more than one successor, giving rise to branching time.

Instead of having to give precise numerical values of the threshold and rate parameters, it is possible to supplement the state equations with inequality constraints on such values. GNA is then able to perform a “qualitative simulation” on the resulting model. Such a simulation results in a state-transition graph consisting of qualitative states and transitions between qualitative states. It is then possible to search for steady states, for example.

#### 4.2.2. Model checking in GNA

GNA is able to perform model checking with NuSMV (Cimatti et al., 2000) and CADP (Garavel et al., 2010). Examples of the kind of properties that can be tested through NuSMV are: “it is possible for a state with an x_a concentration equal to t_xa2 to occur” or “if the system is in the state corresponding to the initial conditions zero_a <= x_a < t_a1, zero_b <= x_b < t_b, and zero_c <= x_c < t_c1, then the system necessarily reaches a steady state.” Importantly, GNA has a pattern-based query language (Monteiro et al., 2008) to help users write CTL formulas.

Through the CADP toolbox, more complex properties can be analyzed (Monteiro et al., 2008). Let A and B be proteins, each of which has an associated threshold. An example of one such property is: “From a given initial state in which A and B have concentrations below their corresponding thresholds, it is possible to reach two different stable states in which only one of A and B is present at concentrations above their corresponding thresholds.” The logic employed (regular alternation-free μ-calculus) subsumes both CTL and propositional dynamic logic (PDL).

In addition, the GNA team has developed computation-tree regular logic (CTRL) (Mateescu et al., 2011), an extension of CTL with regular expressions and fairness operators. CTRL is able to express properties of biological interest and subsumes both CTL and LTL. A particular strength of this logic is the convenient specification of multistability and oscillation properties. CTRL formulae are translated into Hennessy–Milner logic with recursion (an equational variant of the modal μ-calculus), which allows reusing the verification technology available in the CADP toolbox.

GNA has been used (by its developers) for analyzing the GRN controlling the carbon starvation response of *Escherichia coli* (Ropers et al., 2006), as well as for studying the initiation of sporulation in *Bacillus subtilis* (de Jong et al., 2003). Furthermore, GNA has been applied by biology groups. Two examples are: Viretta and Fussenegger (2004) with *Pseudomonas aeruginosa*, and Sepulchre et al. (2007) with a pectinolytic bacterium.

### 4.3. SMBioNet

SMBioNet (Selection of Models of Biological Networks) (Bernot et al., 2004; Richard et al., 2006, 2008, 2012; Khalis et al., 2009) is founded on the formalism developed by Thomas' and his colleagues (Thomas and D'Ari, 1990). The following review of such a formalism is based on (Richard et al., 2008).

#### 4.3.1. Thomas' state-transition systems

Thomas' models of GRNs can be viewed either as an abstraction of a special case of piecewise-linear differential equations or as a generalization of a restriction of Boolean GRNs. These models are multi-valued state-transition systems, where concentrations are represented with discrete variables. In addition, time is viewed as proceeding in discrete steps. The value of every gene *x* at time *t*+1 is specified by a function of the values of its regulators *y*_1_, *y*_2_, … , *y*_*n*_*x*__ at time *t*.

#### 4.3.2. Thomas' method

Thomas and his colleagues developed a method establishing a mapping from an “interaction” graph into a set of multi-valued state-transition systems. An *interaction graph G* is a directed graph where each node corresponds to a gene and each edge *x* → *y* is labeled with a sign. We say that *x* is an *activator* (resp. *inhibitor*) of *y* if the sign is positive (resp. negative). We say that *y* is *influenced* by *x* if there is an edge *x* → *y*.

Thomas' method associates state-transition systems with an interaction graph as follows. First, an instantiated interaction graph is obtained by associating a set of possible “levels” with each gene, and a “threshold” with each edge. Each gene *x* has at most *r*_*x*_ + 1 levels, where *r*_*x*_ is the number of genes influenced by *x*. Intuitively, this allows for the possibility that each gene influenced by a gene *x* reacts at a different threshold of the level of *x*. Hence, with each gene *x* we associate a set of *levels*:

$$S_{x}=\left\{0,\ldots ,r_{x}^{\prime }\right\}$$

where *r*′_*x*_ ≤ *r*_*x*_. In addition, with each interaction *x* → *y* we associate a *threshold*:

$${\text{θ}}_{xy}\in \left\{1,\ldots ,r_{x}^{\prime }\right\}$$

**Figure 2:**
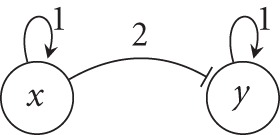
**An instantiated interaction graph where *S*_*x*_ = {0, 1, 2} and *S*_*y*_ = {0, 1}.** An activator is represented by an ordinary arrow; an inhibitor is represented with a blunt arrow.

An example of an instantiated interaction graph is depicted in Figure 2.

The set of possible states is *S* = *S*_1_ × · × *S*_*n*_. The level of *x* at a state *s* = (*s*_1_, … , *s*_*n*_) ∈ *S* is given by the component *s*_*x*_ ∈ *S*_*x*_. The set of *effective regulators* of gene *x* at state *s* is:

$${\text{ω}}_{x}\left(s\right)=\left\{y\in G_{x}^{—\text{1}}|s_{y}\geq {\text{θ}}_{yx}\right\}$$

where *G*^−1^_*x*_ denotes the set of predecessors of *x* in the interaction graph *G*.

Next, Thomas maps each ω_*x*_ (*s*) into an integer *k*_*x*_ (ω_*x*_ (*s*)) ∈ *S*_*x*_ (the value toward which *x* “tends”) as follows. For each gene *x* and for each set of predecessors *R* ⊆ *G*^−1^_*x*_, *k*_*x*_ (*R*) ∈ *S*_*x*_ is an integer satisfying the following *action constrains*:

1. If *y* is an activator of *x*, then
  $$\begin{array}{c} \begin{array}{cc} \forall R\subseteq G_{x}^{-1}, & k_{x}\left(R\right)\leq k_{x}\left(R\cup \left\{y\right\}\right) \end{array} \end{array}$$
2. If *y* is an inhibitor of *x*, then
  $$\begin{array}{c} \begin{array}{cc} \forall R\subseteq G_{x}^{-1}, & k_{x}\left(R\right)\geq k_{x}\left(R\cup \left\{y\right\}\right) \end{array} \end{array}$$

Intuitively, action constraints mean that the action of an activator of *x* cannot decrease the level toward which *x* tends, and that the action of an inhibitor of *x* cannot increase the level toward which *x* tends.

The following table shows, for each state, the effective regulators of each gene, as well as possible values toward which each gene tends.

**Table:** 

*s*_*x*_	*s*_*y*_	ω_*x*_ (*s*)	ω_*y*_(*s*)	*k*_*x*_ (ω_*x*_ (*s*))	*k*_*y*_ (ω_*y*_ (*s*))
0	0	∅	∅	1	1
0	1	∅	{*y*}	1	1
1	0	{*x*}	∅	2	1
1	1	{*x*}	{*y*}	2	1
2	0	{*x*}	{*x*}	2	0
2	1	{*x*}	{*x*}, *y*}	2	1

The *k*'s satisfy the corresponding action constraints:

$$\begin{array}{c} k_{x}\left(\emptyset \right)\leq k_{x}\left(\left\{x\right\}\right)\\ k_{y}\left(\left\{x\right\}\right)\leq k_{y}\left(\emptyset \right)\leq k_{y}\left(\left\{y\right\}\right)\\ k_{y}\left(\left\{x\right\}\right)\leq k_{y}\left(\left\{x,y\right\}\right)\leq k_{y}\left(\left\{y\right\}\right) \end{array}$$

Note that there may be many possible values of the *k*'s satisfying the associated action constraints.

**Figure 3:**
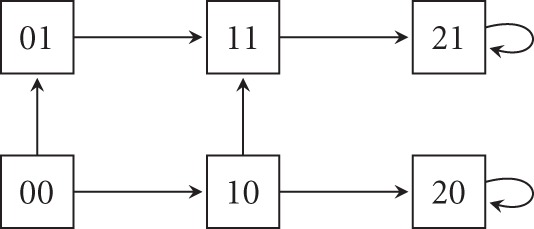
**A state-transition graph of example in Figure 2 showing asynchrony**.

A state-transition system *T* is now obtained as follows. *T* has a transition (edge) from *s* to (*k*_*x*_1__ (*s*), … , *k*_*x*_*n*__(*s*)) if and only if *k*_*i*_ (*s*) ≠ *s*_*i*_ for at most one *i*. Note that a state may have more than one successor. The state-transition system for our example is exhibited in Figure 3.

#### 4.3.3. SMBioNet

**Figure 4:**
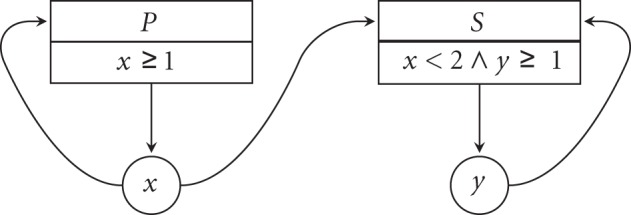
**An interaction graph with processes**.

SMBioNet extends Thomas' formalism with processes. This extension enables the incorporation of biological information possibly constraining the set of state-transition systems associated with an interaction graph. Essentially, processes constrain the regulators of a gene with Boolean functions over inequalities. For example, instead of having *y* independently influenced by *x*, through *Q* if *x* < 2, and by *y*, through *R* if *y* ≥ 1, *Q* and *R* may be combined (e.g., by ∧) into one process *S* to influence *y* if *x* < 2 ∧ *y* ≥ 1 (Figure 4).

SMBioNet takes as input an interaction graph with processes *G* and a temporal property expressed by a CTL formula φ. SMBioNet returns as output all the networks *f* such that the interaction graph (without processes) of *f* is a subgraph of *G*, and the state-transition graph of *f* satisfies φ.

To build the expected output, SMBioNet exhaustively enumerates all the possible values of the sets of parameters and, by using NuSMV, retains the corresponding state graphs satisfying the given CTL formula. Processes and restrictions on the parameters help to reduce the number of parameter sets to be processed.

The number of states for a state graph corresponding to a Boolean network with *n* variables is 2^*n*^. Each of the 2^*n*^ states has at most *n* outgoing transitions (*n* is a condition of Thomas' formalism; the general bound would be 2^*n*^). Since each of the *n*2^*n*^ possible transitions may or may not be present in an asynchronous state graph, the number of asynchronous state graphs for a Boolean network with *n* variables is 2^(*n*2^*n*^)^. Thus, if the input of SMBioNet is a property *p*, and the complete interaction graph has *n* vertices, then the output of SMBioNet consists of all the Boolean networks with *n* components whose asynchronous state graph satisfies property *p*. In this case, SMBioNet complexity is given by 2^(*n*2^*n*^)^. Therefore, SMBioNet only works in general for small values of *n*, typically *n* < 7 (Richard et al., 2012, p. 2).

SMBioNet has been applied to the tail resorption in tadpole metamorphosis (Khalis et al., 2009) and to the immunity control in bacteriophage lambda (Richard et al., 2006).

### 4.4. Pathway logic

Pathway Logic (Eker et al., 2003; Talcott, 2008) employs the Maude computer system (Clavel et al., 2007), based on rewriting logic. In rewriting logic, the behavior of a system is given by local transitions between states, and transitions are described by rewrite rules. A rewrite rule has the form “*t* ⇒ *t*′ *if c*”, where *t* and *t*′ are patterns and *c* a condition. A rule applies to a system in state *s* if *t* can be matched to a part of *s*. Sequences of states transformed through rule application form branching computations (since more than one rule may be applicable).

Similarly to Biocham, the interpretation of a system in Pathway Logic is qualitative and binary (Talcott, 2008, pp. 25, 36).

Pathway Logic can perform forward search (i.e., simulation), as well as LTL model checking (Eker et al., 2003, p. 154) (Talcott, 2008, p. 32) with the LoLA model checker (Schmidt, 2000) for Petri nets. To be model-checked, therefore, a system represented with rewrite rules must be mapped into a Petri net. A Petri net is a directed graph with two kinds of nodes: “transitions” and “places”. When a set of rewrite rules is viewed as a Petri net, the transitions correspond to rewrite rules and the places correspond to reactants, products, or modifiers (Talcott, 2008, p. 40). Unlike other uses of model checking in biochemical network analysis, Pathway Logic can employ model checking to compute paths by means of a counterexample: By giving the model checker a formula asserting that a desired path does not exist, the counterexample computed by the model checker will be precisely such a path.

In addition, Pathway Logic provides metalevel analysis: Rules can be abstracted into families, each family corresponding, for example, to a particular type of reaction, such as activation, inhibition, or translocation. It is thus possible, for instance, to find all rules involving a given protein.

Pathway Logic has been applied to analyze the MAPK pathway (Eker et al., 2003; Talcott, 2008). The model of this pathway has 500 rules, 650 proteins, and 39,992 states (Eker et al., 2003, p. 148).

### 4.5. Antelope

Antelope (Analysis of Networks through TEmporal-LOgic sPEcifications) (Arellano et al., 2011) is a system developed by our team, aimed at Boolean networks. Antelope also uses model checking in a different way from other computational-biology tools. Instead of simply verifying whether or not a given formula holds, Antelope utilizes a model checker to obtain all the states in the model which satisfy a given formula. Hence, if the formula denotes reachability to a state, for example, Antelope will obtain the basin of attraction to such a state.

As with other systems, Antelope has to face the fact that ordinary logics for model checking such as CTL and LTL are not expressive enough for many biological applications. Antelope employs an extension of CTL with “hybrid” operators (not to be confused with “hybrid model checking”, having both discrete and continuous variables). The additional expressiveness of Hybrid CTL essentially consists in formulas being able to refer to particular states explicitly. Hybrid CTL can express many interesting properties such as oscillations and multistability.

Antelope encourages the use of branching time beyond asynchrony, for incompletely specified behavior and environment interaction. The authors exemplify these other uses of branching time in the development of a Boolean GRN of the *Arabidopsis thaliana* root stem cell niche.

### 4.6. Simpathica and XSSYS

Antoniotti et al. (2003) illustrate the use of model checking by Simpathica and XSSYS for qualitative biochemical network analysis. An XS-system is a set of differential equations describing the rate of change of given quantities, together with a set of constraints on these quantities. Simpathica allows the user to enter the description of an XS-system and simulate its behavior. XS-systems may be discretized into state-transition systems which can be model-checked by XSSYS. XSSYS is a model checker employing a variant of LTL in which the propositional variables consist of assertions about the values or rate of change of reactants. This variant of LTL may identify steady states (the rate of change of all concentrations is zero) and express that the concentration of a reactant is within an interval. The authors illustrate their systems with a repressilator system and the purine metabolism in humans.

## 5. Specific uses of model checking

This section is devoted to examples of model checking applied to biochemical networks analysis, without necessarily involving the development of a specialized tool.

### 5.1. Mocha

In (Fisher et al., 2007), the authors follow the idea of using formal verification for testing biological hypotheses. According to the authors, by verifying all possible model executions, the model-checking analysis resembles the study of variations in the rate of biochemical reactions.

The proposal of Fisher et al. (2007) is using model checking for reproducing biological behavior under different mutant backgrounds. Specifically, the authors present a Reactive Modules (Alur and Henzinger, 1999) model describing the *Caenorhabditis elegans* vulval development. Then, the authors use the Mocha model checker (Alur et al., 1998) for querying different behaviors of the model like, for example, the mutations leading to stable or unstable fate patterns. The *C. elegans* analysis of the authors predicts some novel interactions between the model components, and provides a better understanding of the necessary temporal order of events leading to stable cell fate patterns.

Finally, it is interesting to mention the high number of states having many successors. A consequence is the existence of approximately 10^36^ possible executions of the model, and about 92,000 different reachable states. Thus, a simulation approach in this context would not be feasible.

### 5.2. HyTech

In (Ahmad et al., 2006), the authors build upon SMBioNet (Bernot et al., 2004). Ahmad et al. recall that temporal logic can describe some epigenetic properties of GRNs. Thus, after applying Thomas' method, it is possible to use a model checker for selecting GRNs satisfying some desired properties (Bernot et al., 2004). Thomas' discretization process, however, neglects the time delays in the change between genes levels of expression. This loss of information may cause some acceptable GRNs to be discarded during the model-checking selection phase. Particularly, Ahmad et al. show how, in some cases, a discrete model cannot distinguish attractive cycles from unstable steady states. For this reason, the authors propose the use of hybrid modeling of GRNs. Specifically, the authors propose using a subclass of linear hybrid automata (LHA, see Alur and Dill, 1994; Henzinger et al., 1998). The LHA of Ahmad et al. (2006) are discrete state machines with time variables (called “clocks”) and where the dynamics of the variables are governed by linear constraints (with some restrictions on their derivatives). The authors show how to build an LHA model of a GRN considering (linear) delays on gene expressions changes. Such models provide a finer-grained description of GRNs, and are able to distinguish attractive cycles from unstable steady states. Then, by using the command language of the HyTech model checker (Henzinger et al., 1997), the authors provide an algorithm for finding the initial values of the clocks and rates of a GRN model for a given steady state. Finally, Ahmad et al. (2006) exemplify their approach with the *P. aeruginosa* mucus production system.

### 5.3. Prism

We now review three works employing the PRISM model checker (Kwiatkowska et al., 2011). In these three works, model checking is applied to CTMC with CSL. In such CTMCs each transition of the system dynamics is labeled with a rate, which is the parameter of an exponential distribution.

In the first work, starting from a kinetics with non-linear ODEs, Calder et al. (2006) model the RKIP-inhibited ERK pathway with an approach using approximate techniques where concentrations are modeled by discrete abstract quantities. By using PRISM, these authors are able to answer questions such as “what is the probability that if a concentration reaches a certain level, it will remain at that level thereafter?” or “how does varying a given reaction rate affect that probability?”. The size of the model depends on the number *N* of levels of concentration of each species. For example, for *N* = 3, there are 273 states and 1316 transitions; for *N* = 9 there are 28,171 states and 216,282 transitions. A state may have more than one outgoing transition; in that case there is a “race” between such transitions. Calder et al. (2006) list the following advantages of using CTMCs and model checking as compared with simulation: (1) CTMCs allow to model performance, as well as having states with more than one successor, (2) PRISM's high level abstractions enable a separation of system structure from performance, and (3) model checking can compute the probability of a property holding even in the presence of infinitely many paths.

In the next work, Heath et al. (2006) build a model for the FGF (Fibroblast Grow Factor) signaling pathway. These authors demonstrate how several temporal properties, including some with reward-based measures, are applicable to the study of biological systems. In addition, they give several exact and approximate techniques for coping with the state-explosion problem.

The authors' model consists of a PRISM module for each component of the pathway (e.g., FGF, FGFR, Src, etc.), and also a module for each possible compound and receptor residue. Module synchronization allows describing interactions involving multiple elements.

For example, two queries in PRISM are: $P_{=?}\left[{ℱ}^{\left[t,t\right]}a_{grb2:fsr2}\right]$, to find the probability that Grb2 binds with FRS2 at time *t*, and ${ℛ}_{=?}\left[ℱ\left(a_{src\_reloc}\vee a_{a_{p}lc\_\deg}\vee a_{spry\_\deg}\right)\right]$, to find the expected time until the degradation or relocation occurs (where the ℛ represents rewards).

Two classes of state-reduction techniques are described: exact and approximate. The exact approaches group equivalent states in the underlying CTMC, and are (1) “lumpability” (Derisavi et al., 2003), (2) symmetry reduction, and a (3) population-based approach.

The approximate approaches are applicable to networks where the proteins and the receptors have multiple docking sites and engage multiple downstream signaling proteins. The first approximate approach is based on identifying and removing “micro-states” in the network. When the model's reactions differ in orders of magnitude, it is possible to separate “fast” from “slow” reactions. Similarly, it is possible to model molecules' concentrations with abstract quantities, such as “low” and “high.” A final reduction is that of abstraction, involving manual grouping of states.

By using approximate techniques, Heath et al. (2006) reduce a CTMC with 10,285,320 states and 92,767,336 transitions, to one with 80,616 states and 560,520 transitions. Moreover, exact techniques reduce the number of states even more, to 38,661.

In the final work, Ciocchetta et al. (2009) present another use of PRISM, combining probabilistic model checking with stochastic simulation. Model checking is an exhaustive technique, but is subject to the state-explosion problem. Simulation, by contrast, only explores a single trajectory at a time, but is less sensitive to the search-space size. Essentially, Ciocchetta et al. (2009) try to combine the advantages of both these methods by employing a simulator to obtain approximate bounds on the amounts of each species. These bounds mainly determine the size of a system which is subsequently model-checked. Their simulator is based on Gillespie's, employs the Michaelis–Menten kinetics, and models each individual particle. The proposed combination is applied to the control circuit for the λ repressor protein CI of λ-phage in *E. coli*.

## 6. Other specialized techniques

In this section, we survey some systems and algorithms that do not necessarily utilize full-fledged model checking, but are related to model checking because of employing either temporal logic or a symbolic technique.

### 6.1. SeMoCoGRN

Building upon SMBioNet, Fromentin et al. (2007) have developed a method based both on CTL and on constraints, named “Selecting Models by Constraints for GRN.” Like SMBioNet, this method has as input an interaction graph and a CTL formula, and produces as output all networks corresponding to such a graph and satisfying such a formula. Unlike SMBioNet, this method combines Thomas' action constraints with Boolean constraints corresponding to the CTL formula. The resulting constraints are turned to a Java constraint-solver (JaCoP). The authors report their system finding 32 networks among three million possible networks in 800 ms (whereas an exhaustive enumeration took 8.5 min). This experiment was performed in a GRN of *P. aeruginosa.*

### 6.2. Mateus et al.'s system

Another system also using both Thomas' formalism and temporal logic is that by Mateus et al. (2007). Inequalities over the parameters of the model are obtained from the interaction diagram. These inequalities are augmented with LTL formulas specifying desirable properties of the model. The model is traversed forward and paths that do not satisfy the constraints are eliminated, so that only paths satisfying the constraints are retained. This method is illustrated with the mucus production in *P. aeruginosa* and the immunity control in bacteriophage lambda.

### 6.3. GINsim

A system also based on Thomas' formalism is GINsim (Gene Interaction Network simulation) (Chaouiya et al., 2003; Gonzalez et al., 2006; Naldi et al., 2009). As in such a formalism, networks in GINsim have both multi-valued genes and states with more than one successor (representing asynchrony). GINsim employs (Naldi et al., 2007) a symbolic technique, namely BDDs, to compute the set of all stable steady states of a synchronous, Boolean state-transition system. A stable steady state is a single-point attractor having a single successor (itself). Such states are obtained by using BDDs to compute all solutions to:

$$\underset{i}{\Lambda }(x_{i}↔f_{i}(x_{0},\ldots ,x_{n-1}))$$

### 6.4. Dubrova et al.'s sat-based method

There are close connections between Boolean satisfiability (SAT) and Boolean networks (Milano and Roli, 2000; Inoue, 2011), allowing the possibility of computing attractors with SAT solvers. For example, Dubrova et al. (2010) employ a SAT solver to compute the set of attractors (of any size) of a synchronous Boolean network. Essentially, their method works as follows. The accessibility relation of the network is represented by a Boolean formula of the form:

$$T(s,s^{\prime })=\underset{i}{\Lambda }({x^{\prime }}_{i}↔f_{i}(x_{0},\ldots ,x_{n-1}))$$

where *s* = (*x*_0_, … , *x*_*n*−1_) and *s*′ = (x′_0_, … , *x*′_*n*−1_). Next, *T* is composed with itself a number of times giving *T*^*k*^. Any assignment satisfying *T*^*k*^ is a finite path of length *k*. A finite path in which a state is repeated is an attractor. Each time an attractor is found, it is “removed” by adding the negation of a state occurring in such an attractor to the formula to be given to the SAT solver the next time. Dubrova et al. (2010) report, for example, taking 52 ms. to find all seven attractors of a *Drosophila melanogaster* Boolean GRN having 52 genes, where the accessibility relation was composed 52 times. A disadvantage of this method is its being restricted to non-branching synchronous Boolean networks.

## 7. Concluding remarks

We encountered model checking used in numerous and varied ways for biochemical network analysis. This verification technique has been applied to many kinds of biochemical models, ranging from Boolean networks, to Thomas' formalism, to hybrid and timed automata, to CTMC (see Table 1).

**Table 1:** **Summary of modeling tools using model checking**.

**System/Work**	**Model(s)**	**Logic(s)**	**Model checker(s)**
Biocham	ODEs, stochastic, discrete, Boolean	CTL, LTL + num. constr., PLTL	NuSMV, PLTL, violation-degree
GNA	piecewise-linear eq., Boolean	CTL, variant μ-calculus, CTRL	NuSMV, CADP, CTRL
SMBioNet	Thomas'	CTL	NuSMV
Pathway logic	rewrite rules, Petri, Boolean	LTL	LoLA
Antelope	Boolean	Hybrid CTL	Antelope's
Simpathica, XSSYS	ODEs	variant LTL	XSSYS
Fisher et al. (2007)	reactive modules	Alternating-time temp. logic (ATL)	Mocha
Ahmad et al. (2006)	LHA	“while” language	HyTech
Calder et al. (2006)	continuous-time Markov chains	CSL	PRISM
Heath et al. (2006)	continuous-time Markov chains	CSL	PRISM
Ciocchetta et al. (2009)	continuous-time Markov chains	CSL	PRISM

It is clear, on the other hand, that the application of model checkers for biochemical network analysis is still incipient. Many tools we reviewed have only been used by their developers. Two relevant exceptions, however, are Biocham, used e.g., by Bellé et al. (2010) and GNA, used e.g., by Viretta and Fussenegger (2004) and Sepulchre et al. (2007).

In our opinion, there are two situations that could be improved to further the applicability of model checking in this area. First, there is often a mismatch between the kind of model that can be checked and the type of biochemical network model built under uncontrolled conditions, such as the available data. Nevertheless, Biocham and GNA employ two different ways of performing such a link.

Second, writing a formula in the logic underlying the utilized model checker is usually difficult. To be sure, Biocham provides syntactic sugar abbreviating certain common formulas and GNA has a pattern-based query language, but the difficulty persists.

In addition to model checking, there are other computational techniques employed in biochemical network analysis. The potential of simulation and constraint-solving, for instance, should also be assessed. Although perhaps the most direct tool, the relevance of simulation cannot be exaggerated. Constraint-solving, in turn, has been successfully employed, as illustrated in Devloo et al. (2003) and Corblin et al. (2009). Such techniques are not only important in their own right, but more so when they can be combined, for example, with model checking. A system exploiting the advantages of model checking reinforced with simulation is Ciocchetta et al. (2009); systems profited by enriching model checking with constraints are Fromentin et al. (2007) and Rizk et al. (2011).

We devoted this work to biochemical pathways, but model checking has also been applied to other problems in computational biology. An example is (Grosu et al., 2009), where a spatial logic based on quadtrees (Samet, 1984) is used for detecting spiral electric waves in networks of cardiac myocytes.

Our main interest was that of exploring model-checking contributions to biochemical network analysis. We saw, nevertheless, contributions in the other direction as well: the development of model-checking results motivated by biochemical problems. We can mention the update component of Biocham (Chabrier-Rivier et al., 2005), the LTL extensions with constraints and with a measure of the violation degree of a formula (Rizk et al., 2009), and CTRL (Mateescu et al., 2011).

We thus believe that model checking is ready for advancing substantial contributions to biochemical network analysis in particular, and to computational biology in general.

### Conflict of interest statement

The authors declare that the research was conducted in the absence of any commercial or financial relationships that could be construed as a potential conflict of interest.
